# Novel Antioxidants for Animal Nutrition—2nd Edition

**DOI:** 10.3390/antiox15040440

**Published:** 2026-04-01

**Authors:** Matteo Dell’Anno, Luciana Rossi

**Affiliations:** 1 Department of Veterinary Sciences, University of Messina, 98122 Messina, Italy; 2 Department of Veterinary Medicine and Animal Sciences—DIVAS, University of Milan, 29600 Lodi, Italy; luciana.rossi@unimi.it

Oxidative stress is widely recognized as a major biological challenge affecting animal health, productivity, and reproductive efficiency. It arises from an imbalance between the production of reactive oxygen species (ROS) and the capacity of endogenous antioxidant systems to neutralize them. Although moderate ROS production is a normal component of cellular metabolism and signaling, excessive accumulation can damage proteins, lipids, and nucleic acids, ultimately impairing cellular function and physiological homeostasis.

In livestock, oxidative stress is frequently associated with environmental challenges, metabolic demands, nutritional imbalances, and management practices. Conditions such as heat stress, infectious diseases, pregnancy-related metabolic adaptations, and high-performance production levels can significantly increase oxidative pressure. As a consequence, oxidative stress has been linked to reduced growth performance, impaired immune function, reproductive disorders, and decreased product quality in farm animals.

Although extensively studied in livestock species, oxidative stress represents a fundamental biological challenge affecting a wide range of animal taxa, including aquaculture species and companion animals.

In recent years, growing evidence has highlighted the importance of identifying nutritional strategies capable of modulating oxidative status and enhancing antioxidant defenses. Bioactive compounds, plant-derived polyphenols, essential oils, and other functional molecules have demonstrated promising effects in improving animal resilience to oxidative challenges. Moreover, advances in molecular biology have highlighted the role of key regulatory pathways, such as nuclear factor erythroid 2-related factor 2 (Nrf2), in controlling antioxidant responses and maintaining redox homeostasis.

In this context, the current Special Issue aimed to collect recent data addressing the role of functional nutritional strategies to mitigate oxidative stress effects. The contributions included in this Special Issue explored innovative antioxidant strategies in different animal species and production systems, ranging from molecular mechanisms and novel antioxidant carriers to applied nutritional interventions designed to enhance animal health, productivity, and sustainability.

The Special Issue includes a combination of two review papers and five original research articles investigating the role of antioxidant mechanisms and nutritional strategies in different animal species and physiological contexts.

From a thematic perspective, the contributions can be broadly grouped into studies addressing reproductive physiology, nutritional interventions, and environmental stress adaptation.

In their narrative review, Khan et al. (Contribution 1) examine the protective role of bioactive compounds against oxidative stress in mammalian reproductive cells. The authors focus particularly on the Nrf2 signaling pathway. Their review highlights how exposure to xenobiotics and environmental stressors, especially heat stress, can induce oxidative damage and apoptosis in reproductive tissues. Activation of the Nrf2 pathway by natural bioactive compounds enhances the expression of antioxidant enzymes and cytoprotective genes, thereby contributing to the preservation of reproductive cell function. The authors emphasize the potential of targeting the Nrf2 pathway as a promising strategy to mitigate oxidative damage and improve reproductive efficiency in mammals. This work further supports the concept that targeting redox-sensitive pathways represents a promising strategy to improve reproductive efficiency in mammals.

Complementing this perspective, Jin et al. (Contribution 2) explored the emerging role of exosomes as novel regulators of oxidative stress in animal nutrition. Exosomes are small extracellular vesicles involved in intercellular communication and capable of transporting proteins, lipids, and nucleic acids. The authors discuss how these vesicles may contribute to redox regulation by modulating cellular signaling pathways and antioxidant defenses. Compared with traditional antioxidant compounds, exosomes may offer advantages such as improved bioavailability, targeted delivery, and enhanced physiological compatibility. The review highlights the potential of exosome-based strategies as innovative tools for managing oxidative stress and improving animal health and productivity. These findings open new perspectives for the development of next-generation antioxidant strategies based on biological carriers.

In an aquaculture model, Ji et al. (Contribution 3) evaluated the effects of dietary tannic acid supplementation on growth performance, disease resistance, and intestinal health in the Chinese soft-shelled turtle (*Pelodiscus sinensis*). The authors demonstrated that dietary tannic acid improved feed utilization and significantly enhanced antioxidant enzyme activity, including superoxide dismutase, catalase, and glutathione peroxidase. Furthermore, tannic acid supplementation reduced mortality following infection with *Aeromonas hydrophila* and improved intestinal morphology and microbial diversity. Multi-omics analyses suggested that these beneficial effects were associated with modulation of metabolic and immune-related pathways, including the peroxisome proliferator-activated receptor signaling pathway and lipid metabolism.

Similarly, Dey et al. (Contribution 4) investigated the effects of a feeding module containing a blend of garlic oil and cinnamon bark powder in Murrah buffalo calves. Over a 170-day experimental period, supplementation with these phytogenic compounds significantly improved growth rate, feed efficiency, and nutrient digestibility. The treated animals also exhibited enhanced immune responses and increased antioxidant enzyme activities, along with reduced lipid peroxidation. Interestingly, the supplementation also resulted in a substantial reduction in enteric methane emissions, highlighting the dual role of antioxidant-based nutritional strategies in improving both animal performance and environmental sustainability.

The interaction between maternal nutrition, oxidative stress, and offspring development was explored in the study by Escalera-Moreno et al. (Contribution 5) who investigated the effects of maternal hydroxytyrosol supplementation during late gestation in nutrient-restricted beef cows. Hydroxytyrosol, a phenolic compound naturally present in olive-derived products, is widely recognized for its strong antioxidant properties. The authors reported that hydroxytyrosol supplementation improved antioxidant ability and modulated the expression of genes involved in immune regulation and energy metabolism. Notably, these effects extended to the offspring, indicating that maternal antioxidant supplementation may influence fetal programming and early-life physiological adaptations.

Another study focusing on reproductive outcomes was conducted by Pesántez et al (Contribution 6), who investigated the effects of supplementing underfed pregnant sheep with polyphenols derived from olive and grape extracts. The results showed that antioxidant supplementation increased maternal total antioxidant capacity and improved metabolic status during gestation. Importantly, the treatment significantly increased the birth weight and size of lambs, particularly in twin pregnancies, which are typically associated with higher metabolic demands and oxidative stress. These findings suggest that plant-derived polyphenols may represent a valuable nutritional strategy to support reproductive performance under challenging environmental or nutritional conditions.

Finally, Nanto-Hara and Ohtsu (Contribution 7) examined the effects of dietary 5-aminolevulinic acid (ALA) supplementation in laying hens exposed to chronic heat stress. Heat stress is a major challenge in poultry production, leading to reduced productivity and physiological dysfunction. The authors demonstrated that ALA supplementation alleviated heat stress-induced renal damage and improved egg production and eggshell quality. At the molecular level, ALA enhanced mitochondrial function and activated antioxidant pathways, including the upregulation of NRF2 and heme oxygenase-1 expression. These results reinforce the relevance of targeting mitochondrial function and redox balance to improve resilience under environmental stress conditions. Collectively, the contributions included in this Special Issue provide valuable insights into the complex relationship between oxidative stress, animal physiology, and nutritional interventions. The studies further confirm that oxidative stress plays a crucial role in a wide range of biological processes affecting animal health, reproduction, productivity, and resilience to environmental challenges.

Importantly, the findings highlight the potential of diverse nutritional strategies based on antioxidants. However, despite these promising results, several challenges remain, including the need for standardized dosages, a deeper understanding of species-specific responses, and the validation of long-term effects under commercial farming conditions. Nevertheless, these compounds may represent a valuable strategy to modulate oxidative stress and enhance animal health and performance.

Considering that most animal diseases are multifactorial in origin, involving interactions among host-, environment-, and pathogen-related factors, antioxidant-based nutritional strategies may contribute to reducing the need for antibiotic interventions by mitigating oxidative stress, which plays a key role both in the onset and progression of pathological conditions, thereby supporting a salutogenic approach to animal health and contributing to the global effort to limit antimicrobial resistance. From a One Health perspective, improving oxidative resilience through nutrition may have implications beyond animal production, contributing to safer food systems and reduced environmental impact.

A major challenge lies in the standardization of antioxidant sources, dosages, and delivery systems, which currently hinders both the comparability of results and their effective translation into practical applications.

Advances in molecular and omics technologies further contribute to a deeper understanding of the basic physiological mechanisms of antioxidant responses, enabling the development of more targeted and effective nutritional approaches.

Future research should continue to explore the interactions between diet, oxidative stress, and animal physiology, with particular attention to species-specific responses, molecular mechanisms, and long-term impacts on productivity and sustainability. Such efforts will be essential to support the development of innovative strategies aimed at improving animal welfare, production efficiency, and environmental sustainability in modern livestock systems.

